# A Case of Choriocarcinoma Undergoing Laparoscopic Surgery Due to Suspected Peritoneal Pregnancy

**DOI:** 10.7759/cureus.86529

**Published:** 2025-06-22

**Authors:** Takuto Maekawa, Senn Wakahashi, Kenta Obata, Koki Moriuchi, Yutoku Shi, Yuki Sasagawa, Satoshi Nagamata, Masashi Nishimoto, Motoyoshi Maruo, Yoshito Terai

**Affiliations:** 1 Department of Obstetrics and Gynecology, Kobe University Hospital, Hyogo, JPN; 2 Department of Obstetrics and Gynecology, Hyogo Prefectural Tamba Medical Center, Hyogo, JPN

**Keywords:** extrauterine choriocarcinoma, gestational trophoblastic neoplasia, laparoscopy, peritoneal pregnancy, pituitary hcg

## Abstract

Extrauterine choriocarcinoma is uncommon and may be mistaken for a ruptured ectopic pregnancy. Rapid diagnosis is essential, as massive intraperitoneal bleeding can be fatal. Once histologically diagnosed, choriocarcinoma is highly chemosensitive.

This case describes a 38-year-old woman who presented with sudden lower abdominal pain. Six weeks after her last menstrual period, serum human chorionic gonadotropin (hCG) was 28,834 mIU/mL. No intra-uterine gestational sac was found, and intraperitoneal bleeding was observed, suggesting an ectopic pregnancy. An emergency laparoscopic surgery revealed a blood clot and active bleeding on the peritoneal surface near the ileocecal region, which was resected. Histology revealed sheets of syncytiotrophoblasts and intermediate trophoblast cells without villi, and immunohistochemistry was diffusely positive for Ki-67 and hCG, confirming primary peritoneal choriocarcinoma. Staging imaging revealed no other lesions. The patient received four cycles of MEA chemotherapy (methotrexate, etoposide, actinomycin D) at three-week intervals, resulting in sustained hCG normalization and no evidence of recurrence at follow-up.

Primary peritoneal choriocarcinoma should be considered in the differential diagnosis of intraperitoneal bleeding during early pregnancy. Even when ectopic pregnancy is suspected, the excised tissue must be submitted for histopathological examination so that chemotherapy can be initiated promptly in case of choriocarcinoma.

## Introduction

Choriocarcinoma can occur after any event during pregnancy: 50% after molar pregnancy, 25% after miscarriage or tubal pregnancy, and 25% after full-term or premature birth. The incidence of choriocarcinoma is approximately 3 cases per 100,000 births in Europe and North America but approximately 23 cases per 100,000 births in Southeast Asia. The risk increases with maternal age [1,2]. The definitive diagnosis of choriocarcinoma was based on the histological examination. However, in extrauterine choriocarcinoma, no lesions are found in the uterus, and there are no symptoms, such as abnormal uterine bleeding, making it difficult to suspect choriocarcinoma. It is often discovered because of symptoms or bleeding of metastatic lesions, such as pulmonary hemorrhage, gastrointestinal hemorrhage, intraperitoneal hemorrhage, and cerebral hemorrhage, and histological diagnosis is not easy. The clinical presentation is similar to ectopic pregnancy, so some rare pelvic lesions may be misdiagnosed as ectopic pregnancy [3]. Therefore, even in cases of suspected ectopic pregnancy, pathological diagnosis is important, and the possibility of choriocarcinoma must be considered and treated [3]. Here, we report a case in which laparoscopic surgery was performed for a suspected peritoneal pregnancy, and the pathological diagnosis was choriocarcinoma.

## Case presentation

The patient was a 38-year-old woman, gravida 3, para 2 (2 full-term births, 1 induced abortion). Her previous pregnancy was a full-term birth at 29 years old. There was nothing to note in her medical or family history.

She visited our hospital complaining of lower abdominal pain. She tested positive for pregnancy and was thought to be at six weeks since her last menstrual period. Ultrasound findings showed no gestational sac in the uterus, but fluid retention suggestive of blood clots was observed in the Douglas and vesicouterine pouches. Laboratory findings are shown in Table 1.

**Table 1:** Laboratory values WBC: white blood cell; RBC: red blood cell; Hb: hemoglobin; PLT: platelet; AST: aspartate aminotransferase; ALT: alanine aminotransferase; LD: lactate dehydrogenase; ALP: alkaline phosphatase; BUN: blood urea nitrogene; CRE: creatine; hCG: human chorionic gonadotropin

Parameter	Value	Reference range
WBC	8,360	3,300-8,600 /μL
RBC	4.73×10^6^	3.86-4.92×10^6^ /μL
Hb	11.9	11.6-14.8 g/dL
PLT	21.7×10^4^	15.8-34.8×10^4^/μL
AST	11	13-30 U/L
ALT	9	7-30 U/L
LD	142	124-222 U/L
ALP	35	38-113 U/L
BUN	8.4	8-20 mg/dL
CRE	0.75	0.46-0.70 mg/dL
hCG	28,834	mIU/ml

**Figure 1:**
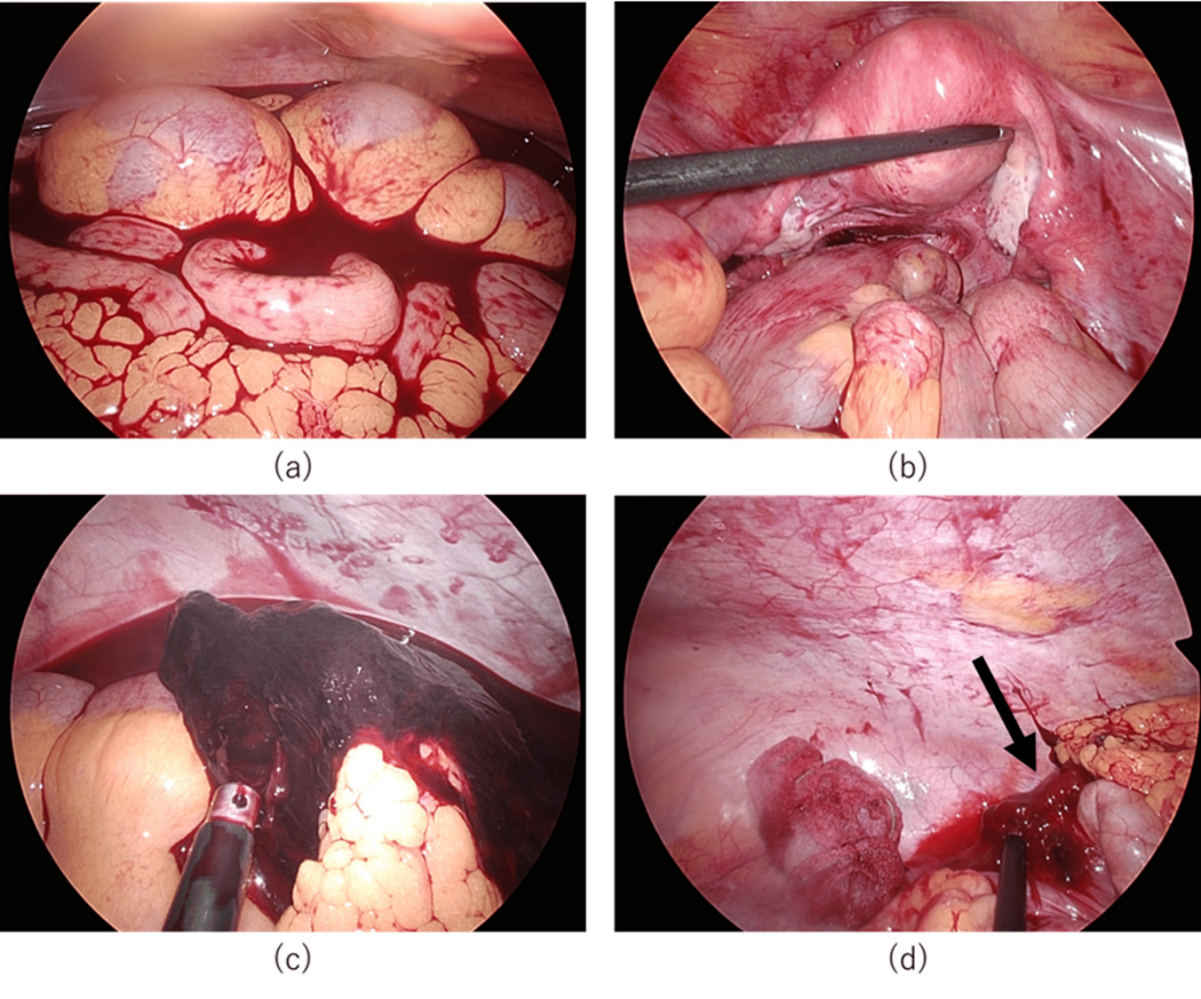
Intra-abdominal findings at laparoscopic surgery (a) Significant bloody ascites. (b) No abnormal findings were found in the uterus or bilateral adnexa. (c) A 20 mm blood clot was found near the ileocecal area. (d) Active bleeding was observed from outside the ascending colon near the ileocecal area.

An ectopic pregnancy was suspected, and emergency laparoscopic surgery was performed. Observation of the abdominal cavity revealed significant bloody ascitic fluid. No abnormal findings were observed in the uterus or adnexa. A 20 mm blood clot was found near the ileocecal area. After the clot was removed, active bleeding was observed from outside the ascending colon near the ileocecal area. This area was considered to be the site of peritoneal pregnancy; the ileocecal area was mobilized, and the affected area was excised and removed from the body. The operative time was 4 h and 13 min, respectively. Intraoperative blood loss was 1450 ml (Figure 1).

**Figure 2:**
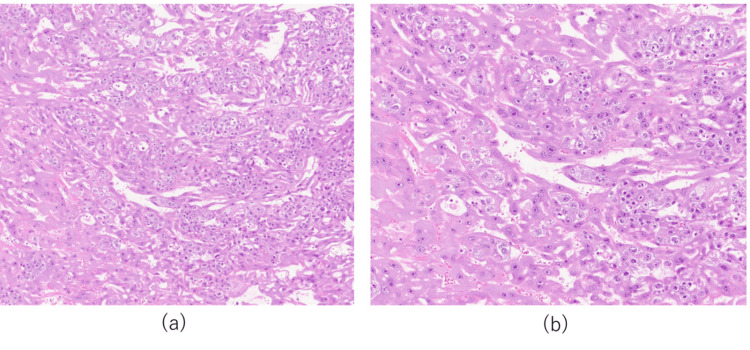
Histopathological examination of the resected tissue (H&E staining) (a) 200×magnification; (b) 400×magnification; (a–b) Histology of the excised tissue showing the proliferation of syncytiotrophoblast cells and intermediate trophoblast cells

The pathological findings of the excised tissue showed a large amount of blood clots, proliferation of acidophilic multinucleated syncytiotrophoblast cells, and proliferation of intermediate-type trophoblast cells with round nuclei and clear cytoplasm. In addition, no villous structures were observed, and based on these findings, the diagnosis was choriocarcinoma (Figure 2) [4].

**Figure 3:**
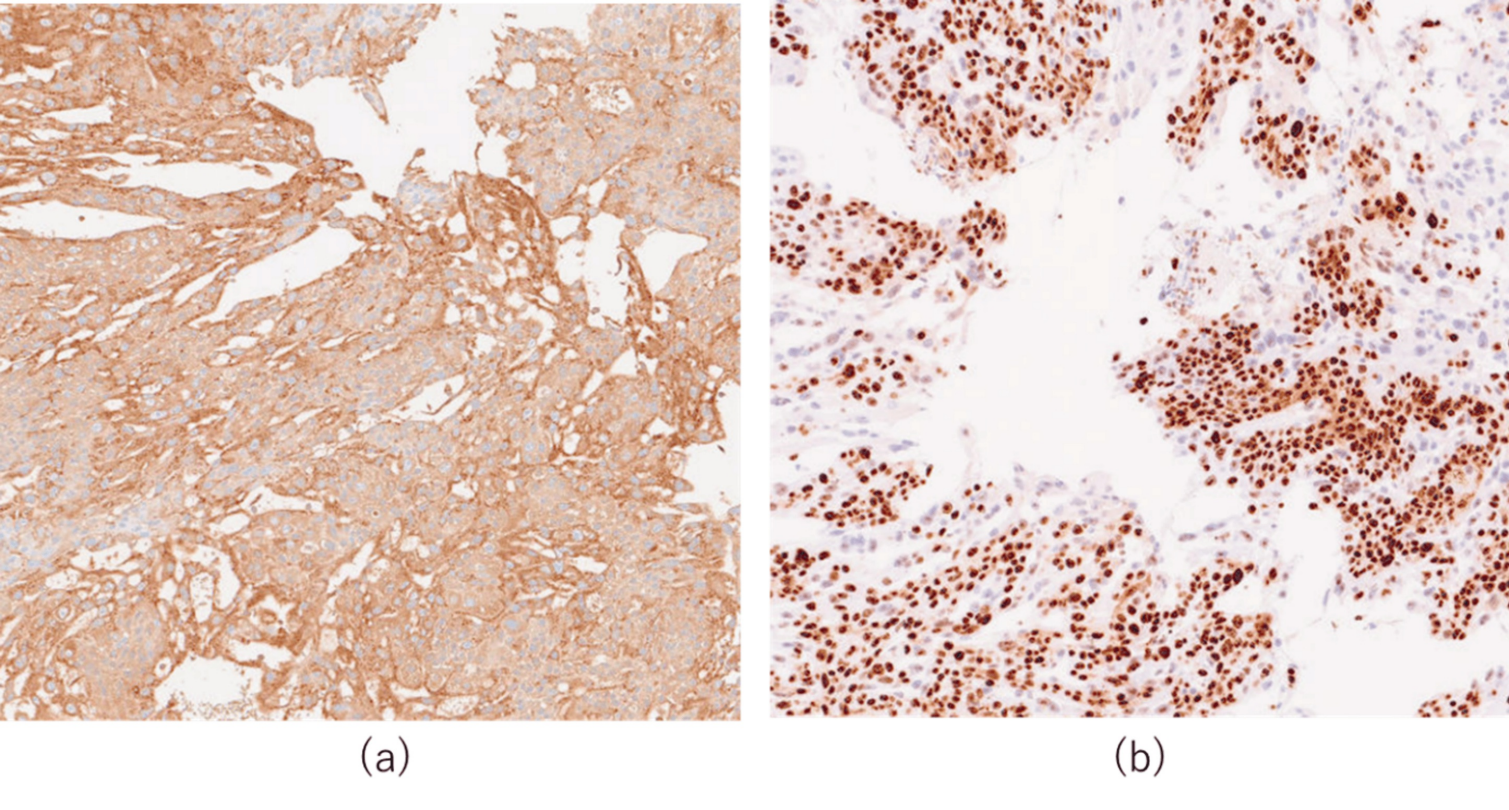
Immunohistochemical staining for hCG and Ki-67 (a-b) 200×magnification; (a-b) Histology of the excised tissue showing positivity for (a) HCG and (b) Ki67 in syncytiotrophoblast cells hCG: human chorionic gonadotropin

HCG and Ki67 staining was diffusely positive in syncytiotrophoblast cells, which supports the diagnosis of choriocarcinoma (Figure 3) [4,5].

**Figure 4:**
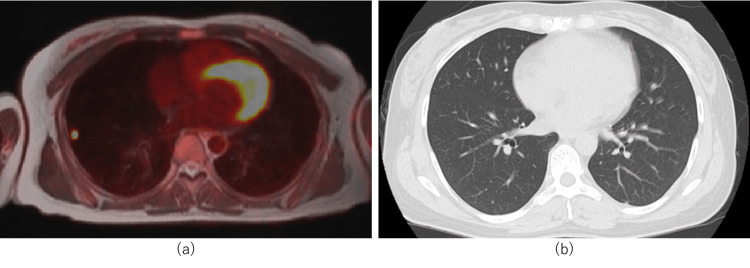
PET-CT and contrast CT of the lung (a) A positron emission tomography (PET)-CT scan revealed a nodule with a high uptake in the right lung; (b) a contrast CT scan revealed no obvious nodule in the right lung

The pathological diagnosis was choriocarcinoma. A positron emission tomography (PET)-CT scan revealed a nodule with high uptake in the right lung; however, a subsequent contrast CT scan revealed no obvious nodule in the right lung (Figure 4).

**Figure 5:**
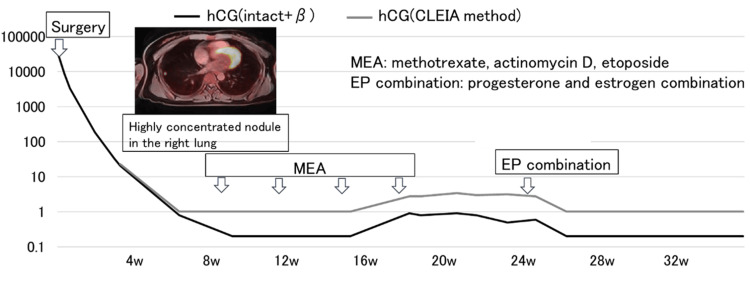
Treatment progress and serum hCG levels Serum hCG levels during MEA therapy and subsequent EP therapy. Low-level hCG elevation after the third MEA cycle suggested a pituitary origin, which resolved following EP therapy. hCG: human chorionic gonadotropin; MEA: methotrexate, etoposide, actinomycin D; EP: luteinizing and estrogen hormones

In addition, no findings suggestive of distant or lymph node metastasis were found. MEA therapy (methotrexate 450 mg/body on day 1, etoposide 100 mg/body on days 1-5, and actinomycin D 0.5 mg/body on days 1-5) was initiated. Four courses of MEA therapy were administered every three weeks. hCG levels remained negative; however, after the third course of MEA, low-unit hCG levels persisted. Considering the possibility of pituitary hCG based on estradiol (E2), follicle-stimulating hormone (FSH), and luteinizing hormone (LH) levels, a combination of luteinizing and estrogen hormones (EP combination) was administered, and the hCG level was negative (Figure 5).

Thereafter, follow-up observation was continued, and no recurrence was observed.

In some cases, low levels of hCG persist without increasing after treatment for gestational trophoblastic neoplasia, which may be due to pituitary hCG levels. In this case, it was thought that there was a high possibility of pituitary hCG; therefore, a combination of progesterone and estrogen was administered, and the hCG level became negative. If low levels of hCG persist, invasive mole or choriocarcinoma may be suspected, and chemotherapy may be considered, but the possibility of pituitary hCG must be considered [1,4,6].

## Discussion

The peritoneum is a rare site for primary choriocarcinoma development. Only nine reported cases of primary peritoneal choriocarcinoma have been reported [7].

Choriocarcinoma is primarily diagnosed by histological examination, but extrauterine choriocarcinoma is difficult to detect because there are no lesions in the uterus, and symptoms are unlikely to appear [8]. Uterine choriocarcinoma has lesions in the uterus, symptoms such as abnormal uterine bleeding are present, and histological diagnosis is possible; however, extrauterine choriocarcinoma has lesions in areas where ectopic pregnancy can occur; no lesions are found in the uterus, and there is no abnormal uterine bleeding, making it difficult to suspect the onset of choriocarcinoma. It is often discovered because of symptoms or bleeding from metastatic lesions, and a histological diagnosis is difficult [8]. When a diagnosis cannot be made by tissue testing, it is made using scoring such as the FIGO (International Federation of Gynecology and Obstetrics) risk score. However, there is a risk that the FIGO risk score will not result in a diagnosis of clinical choriocarcinoma [9]. Without a tissue diagnosis, there was a high possibility that the hCG levels would have continued to be monitored; therefore, obtaining a tissue diagnosis made it possible to intervene early. Choriocarcinoma is treated well with chemotherapy, and with MEA therapy, the initial remission rate is 75-88%, with a recurrence rate of 4-10%.

Peritoneal pregnancy can cause heavy bleeding, and in this case, it was difficult to confirm the location of the pregnancy and stop bleeding [10]. Extrauterine choriocarcinoma of primary peritoneal origin is rare, but in cases where ectopic pregnancy is suspected, accompanied by intraperitoneal bleeding, careful observation of the abdominal cavity and pathological diagnosis are considered important.

In cases in which ectopic pregnancy is suspected, pathological diagnosis is important, and the possibility of choriocarcinoma should be considered. It is also important to keep in mind that choriocarcinoma can occur secondary to pregnancy.

## Conclusions

Choriocarcinoma occurs in a small percentage of cases of hydatidiform mole, but it can occur after any pregnancy, including normal delivery and miscarriage. In this study, we observed cases in which laparoscopic surgery was performed for suspected ectopic pregnancy, and intraoperative findings suggested a peritoneal pregnancy, but postoperative pathological diagnosis revealed choriocarcinoma.
